# Demographic outcomes of diverse migration strategies assessed in a metapopulation of tundra swans

**DOI:** 10.1186/s40462-016-0075-8

**Published:** 2016-05-01

**Authors:** Craig R. Ely, Brandt W. Meixell

**Affiliations:** U.S. Geological Survey, Alaska Science Centre, 4210 University Drive, Anchorage, AK 99508 USA

**Keywords:** *Cygnus columbianus*, Known fate, Life history, Metapopulation, Migration distance, Productivity, Satellite telemetry, Survival, Transmitter effects, Tundra swan

## Abstract

**Background:**

Migration is a prominent aspect of the life history of many avian species, but the demographic consequences of variable migration strategies have only infrequently been investigated, and rarely when using modern technological and analytical methods for assessing survival, movement patterns, and long-term productivity in the context of life history theory. We monitored the fates of 50 satellite-implanted tundra swans (*Cygnus columbianus*) over 4 years from five disparate breeding areas in Alaska, and used known-fate analyses to estimate monthly survival probability relative to migration distance, breeding area, migratory flyway, breeding status, and age. We specifically tested whether migratory birds face a trade-off, whereby long-distance migrants realize higher survival rates at the cost of lower productivity because of reduced time on breeding areas relative to birds that migrate shorter distances and spend more time on breeding areas.

**Results:**

Annual migration distances varied significantly among breeding areas (1020 to 12720 km), and were strongly negatively correlated with time spent on breeding areas (*r* = −0.986). Estimates of annual survival probability varied by wintering area (Pacific coast, Alaska Peninsula, and Eastern seaboard) and ranged from 0.79 (95%CI: 0.70–0.88) to 1.0, depending on criteria used to discern mortalities from radio failures. We did not find evidence for a linear relationship between migration distance and survival as swans from the breeding areas with the shortest and longest migration distances had the highest survival probabilities. Survival was lower in the first year post-marking than in subsequent years, but there was not support for seasonal differences in survival. Productivity varied among breeding populations and was generally inversely correlated to survival, but not migration distance or time spent on breeding areas.

**Conclusions:**

Tundra swans conformed to a major tenet of life history theory, as populations with the highest survival generally had the lowest productivity. The lack of a uniform relationship between time spent on breeding areas and productivity, or time spent on wintering areas and survival, indicates that factors other than temporal investment dictate demographic outcomes in this species. The tremendous diversity of migration strategies we identify in Alaskan tundra swans, without clear impacts on survival, underscores the ability of this species to adapt to different environments and climatic regimes.

## Background

Migration is a behavioural characteristic that is thought to have evolved to maximize fitness in seasonal environments, and is a prominent aspect of the life history of many avian species [1–4]. Understanding the demographic consequences of migration has long been a goal of avian ecologists, as identifying the fitness costs associated with different behaviours should further our understanding of mechanisms driving the evolution and maintenance of avian life history strategies [5, 6]. For arctic-breeding birds undergoing lengthy biannual migrations, the costs of such an energy demanding behaviour may be substantial in terms of both reproduction and survival [3, 7, 8].

The energy cost of long migrations is undeniably high with potentially large negative impacts on reproduction, as migrants must balance between using energy for flight and storing reserves for reproduction [9, 10]. Such costs are likely greatest in pure capital breeders, that unlike income breeders, cannot offset the energetic burden of migration by foraging on breeding areas. The cost of migration, especially long-distance migration, on survival is less clear but of great interest, because the population dynamics of long-lived species are generally predicated on adult survival [11, 12].

There are several competing hypotheses concerning the demographic consequences of long distance migration. Many authors have argued that long migrations must exact a toll in terms of reduced survival compared to sedentary species [7, 13], and it has been shown that birds carrying more energy reserves (and thus capable of flying farther) are more vulnerable to predation than leaner birds [3, 14]. Birds with longer migrations may also suffer increased mortality because they cross a greater diversity of landscapes and are hence potentially exposed to a larger suite of predators than sedentary species. In contrast, others have argued that birds migrate to areas where mortality is reduced, and are therefore expected to have higher survival rates than resident species or species that travel less far [15–18]. In fact, Greenberg [15] proposed that migratory birds face a trade-off, whereby long-distance migrants realize higher survival rates because they travel further to reach more benign wintering areas, but at the cost of lower productivity due to spending concomitantly less time on breeding areas [15, 19]. He termed this dichotomy of investment the “Time Allocation” hypothesis which predicts that, in migratory birds, productivity and survival are dictated by temporal investment, with any increase in length of the breeding season leading to a decrease in the amount of time for occupying the non-breeding range. As such, one prediction is that populations at higher latitudes, with long migration distances, should have higher survival rates and lower productivity than residents or short-distance migrants [15, 20]. Several studies have tested predictions of the time allocation hypothesis with variable results (see reviews in [19, 21, 22]).

One reason for the persistence of such differing theories attempting to explain the inter relationships among migration distance, survival, and productivity is the lack of suitable quantitative data, especially across populations of a single species (i.e., a metapopulation). Intra-specific comparisons are necessary to test a more rigorous theoretical framework for weighing life-history trade-offs, as cross-species comparisons may be misleading because selective forces act differently on taxa with different life history characteristics. Nichols [21], in a review of survival rates relative to migration distance, found equivocal results among studies [15, 20, 23–25] of which he partially attributed to the fact that many studies used >1 species, and used unreliable methods to determine survival rates. Nichols [21] went on to specifically note how informative it would be to “estimate fitness components (survival and reproductive rates) for animals associated with different breeding or wintering habitats of a migratory metapopulation.”

Identifying seasonal timing of mortality in migratory birds is also a key consideration, as estimates of annual survival, while useful, preclude attributing mortality to a specific period of the annual cycle and may limit identification of factors likely regulating the population. In one of the few studies to link survival of long-distance migrants to seasonal time periods, Klaassen et al. [13] monitored the migration of several species of raptors using satellite telemetry, and found that morality rates were indeed highest during periods of migration.

Tundra swans (*Cygnus columbianus*) are large migratory waterfowl that breed in tundra habitat throughout most of the Holarctic and winter in north temperate climates at varying degrees of latitude. The North American subspecies (*C. c. columbianus*) nests nearly continuously from the tip of the Alaska Peninsula in south-western Alaska to the east side of Hudson Bay Canada, as well as in far eastern Chukotka [26]. Migration is a prominent behaviour of tundra swans, as they spend most of the year migrating to and from the breeding grounds. Migration distances and routes vary considerably among often disjunct wintering populations [27–29], allowing for detailed assessment of demographic parameters relative to variation in migratory behaviour. Here, using modern technological and analytical methods, we present information on factors related to the survival of tundra swans implanted with satellite transmitters that migrated variable distances to wintering sites from five different breeding areas across their range in Alaska. We use an information-theoretic approach and known fate survival estimation to assess the relative importance of factors likely influencing the survival of swans, including migration distance, migratory flyway, age, and breeding success, while accounting for disease (prevalence of avian influenza viruses and blood parasites) and contaminants. We compare survival estimates and breeding-area-specific indices of productivity to assess potential trade-offs between survival and productivity in the context of migration distance and temporal investment.

## Methods

### Study species

**Fig. 1:**
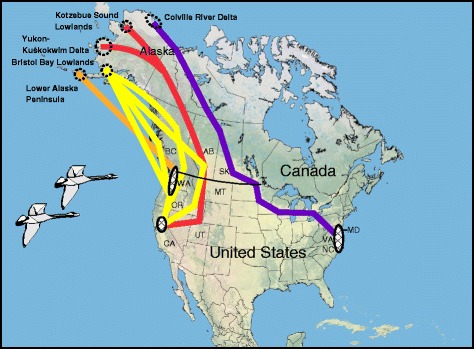
Location of satellite-marking of tundra swans in Alaska during 2008, showing primary migration routes from five different breeding areas. Autumn and spring migration routes are similar. Hatched polygons are wintering areas. Most Lower Alaska Peninsula swans do not migrate

Tundra swans (*Cygnus columbianus*) are considered a k-selected species as they have delayed reproduction (do not breed until at least 3 years of age), and are known to live over 20 years in the wild [26]. Tundra swans are highly reliant on wetlands for roosting and feeding, but have also adapted to feeding on agricultural crops [30]. For our study, tundra swans were captured when flightless, during the annual wing molt in July and August, 2008 at five different breeding areas in Alaska (Fig. 1). The sex of birds was determined based on cloacal characteristics and internal examination. Birds were classified to age as either locals (i.e. cygnets), second year birds (SY; birds hatched the previous year), or after second year birds (ASY). Second year birds were distinguished from older birds by the presence of gray feathering on the head and neck, and sometimes, back [30]. Swans were categorized relative to breeding status as either breeders (adults with cygnets), territorial pairs (adults without cygnets on a territory), or non breeders (ASY or SY birds in groups). Satellite transmitters (platform transmitting terminals [PTTs]; model 100, two AA batteries [50 g total mass], Microwave Telemetry, Columbia, MD); were abdominally implanted [31] in 50 tundra swans (ten at each of five different breeding areas) throughout the range of the species in Alaska. Transmitters had percutaneous antennas [32, 33] that exited the body to provide the signal strength necessary for satellite transmission.

### PTT duty cycles and processing location information

PTTs were programmed to transmit for 5 h and then turn off for 72 h during summer and winter periods, and turn off for 18 h during migration periods in autumn and spring [34]. The PTTs were designed to transmit for 2 years; if the 2-year period was exceeded, the duty cycle reverted to 5 h on and 72 h off for the life of the transmitter. The Argos Data Collection and Location System [35] was used to obtain information on latitude and longitude, date, time, quality of location, body temperature, and activity of swans instrumented with PTTs. Unlikely locations were filtered based on rate and angle of movement and the highest quality locations were used to represent daily position [36].

### Detection of disease and contaminants

The presence of low pathogenic avian influenza (LPAI) viruses in tundra swans can affect migration behaviour [37], so birds were tested for active LPAI viruses at the time of capture. Cloacal and oral-pharyngeal (OP) swabs were obtained and either analyzed individually or pooled in the field or in the laboratory in groups of 2 to 5 by sampling location. Detection of birds actively shedding AI viruses was determined according to the standardized USDA National Animal Health Laboratory Network AI real time reverse transcriptase polymerase chain reaction (RT-PCR) protocol [38–40]. Previous work summarized levels of blood parasite infection [34] and lead contamination [41] in PTT-marked and unmarked birds from each population.

### Migration distance and time on breeding areas

Great circle migration distances were calculated using the ruler function in Google Earth to directly measure between stopover locations for each PTT-marked swan. Annual migration distances were determined by summing the distance flown by each PTT-marked bird from the breeding location to the southernmost wintering area in the autumn, with the distance flown from the wintering area back to the breeding area the following spring. Time on breeding areas was calculated by subtracting the date the PTT-marked swan arrived on the breeding area in the spring from the date it left the area the following autumn. If birds did not migrate away from the breeding ground they were considered breeding ground residents for 365 days per year. If location information was not received from a PTT on the date of arrival or departure, then the date of arrival or departure was assumed to be the midpoint between the dates when the PTT-marked swan was known to be at the wintering or breeding site and the previous or succeeding date, when it was enroute. Differences in distance migrated by swans from the different breeding areas, and time spent on breeding areas, were assessed with analysis of variance using a mixed model (PROC MIXED, [42]) to accommodate random and fixed effects. A repeated measures design was implemented to accommodate migration distances flown by each bird across 3 years, and least square means was used for post hoc pairwise comparisons. Mean estimates of annual migration distance for swans from each breeding area were used as covariates in survival analysis.

### Survival analysis

Known-fate modeling was used in Program MARK [43] to estimate monthly survival probabilities (MSP) of PTT-marked tundra swans. Interval lengths corresponded to calendar months, beginning in August of 2008 and ending in July of 2012. In each month, birds were specified as alive, dead, or censored based on information received from the transmitters. Swans were assumed to be alive in a given interval if the transmission in that month revealed normal body temperature (>40 °C) and normal battery voltage (>3.2v). Birds were considered to have died during an interval if sensors indicated a drop in body temperature while battery voltage remained above 3.2v. Months in which the fate of birds were unknown (i.e., signals not received from transmitters) were censored and therefore did not contribute data. It was assumed that a transmitter failed while a bird was still alive if sensors indicated normal body temperature and the battery voltage declined below 3.2v; encounter histories of birds with transmitter failures were censored to the previous interval of known fate. Discrepancies in determining fate occurred when the final transmission revealed normal body temperature and normal battery voltage. Under this scenario, it was unclear whether 1) the bird died in an event that damaged or otherwise blocked the transmitter signal, or 2) the transmitter failed due to circuitry, battery, or antenna issues and the bird remained alive. An assessment of location and activity data did not reveal information that reconciled these discrepancies, and we suspected that any of the options were plausible. Therefore, our analyses are based on two versions of the data following methods similar to Hupp et al. [44]. In the first analysis, a conservative measure of mortality was used in which ‘discrepancies’ were treated as radio failures, and their encounter histories were censored to the interval prior to transmitter failure. In the second analysis, a more liberal approach was used for interpreting mortalities by treating discrepancies as mortalities in the interval the transmitter went off the air.

Encounter histories were constructed with breeding location as groups (Lower Alaska Peninsula [LAP], Bristol Bay Lowlands [BBL], Yukon-Kuskokwim Delta [YKD], Kotzebue Sound Lowlands [KSL], and Colville River Delta [CRD]), a breeding-area specific covariate of annual migration distance, and the individual covariates of age and breeding status. For both versions of the capture histories, an initial suite of 22 models was considered that assessed variation in MSP relative to migration distance, location (breeding and wintering areas, and management population), time (year, season, season*year), age and breeding status, and transmitter effects (Tables 1 and 2). Because our initial model set did not contain combinations of some variables, additional models were constructed that represented combinations of variables from models that were supported in the initial model set.

**Table 1:** Characteristics of breeding populations of tundra swans implanted with satellite transmitters relative to marking location in Alaska. Refer to Fig. 1 for detailed distribution information

Marking location	Management population	Migration route	Winter location	Annual migration distance ± SE (km)	Time spent on breeding area ± SE (d)	Hunter harvest
Colville River Delta	Eastern	Upper Plains	NC, MD,VA	12719 ± 119	115.2 ± 2	yes
Kotzebue Sound Lowlands	Western	AB, SK, MT, UT	CA	10753 ± 85	141.1 ± 3	yes
Yukon-Kuskokwim Delta	Western	AB,SK, MT, UT	CA	10963 ± 133	130.9 ± 4	yes
Bristol Bay Lowlands	Western	seAK, BC	Pacific NW, CA	7906 ± 286	172.6 ± 5	min.
Lower Alaska Peninsula	Western	Gulf of Alaska	AK,WA, BC	1021 ± 405	351.5 ± 7	no

Annual migration distance was the combined distance of autumn and spring migration to and from marking locations on the breeding area to primary wintering location. Hunter harvest of Bristol Bay tundra swans was considered minimal as they do not commonly migrate through the western states of Montana, Idaho and Utah, that allow sport harvest of tundra swans, and there is no sport harvest for tundra swans allowed in the Bristol Bay region of Alaska. Tundra swans from the Lower Alaska Peninsula are facultative migrants; only 2 of the 10 PTT-marked swans from the Lower Alaska Peninsula migrated during the study, and only irregularly

**Table 2:** A priori models (*n* = 22) considered for known-fate estimation of monthly survival probabilities (MSP) of PTT-implanted tundra swans from Alaska, USA, 2008–2012. Ten swans were marked and released at each of 5 breeding areas: Lower Alaska Peninsula (LAP), Bristol Bay Lowlands (BBL), Yukon-Kuskokwim Delta (YKD), Kotzebue Sound Lowlands (KSL), and Colville River Delta (CRD). Year 1 refers to the 12 months following release in August of 2008. K = number of model parameters

MSP model	K	Model description
Location models		
S(Migration distance)	2	Survival varies relative to annual migration distance
S(Management population)	2	Survival varies by Western Population (LAL, BBL, YKD, KSL) and Eastern Population (CRD)
S(Wintering area)	3	Survival varies by location of wintering area (BBL, YKD, KSL + LAL + CRD)
S(Breeding area)	5	Survival varies by 5 breeding areas (LAL + BBL + YKD + KSL + CRD)
Time models		
S(.)	1	Survival is constant
S(Yr)	4	Survival varies by year
Season models		
S(Migration)	2	Survival during migration seasons (Autumn, Spring) varies from sedentary seasons (Winter, Breed)
S(Autumn)	2	Survival during Autumn varies from other seasons (Spring, Winter, Breed)
S(Season)	4	Survival varies by 4 seasons (Spring + Autumn + Winter + Breed)
Age and breeding status models		
S(Age*Yr1 + Yrs2-4)	3	Survival varies between SY and ASY birds in Year 1 and is constant thereafter
S(Breeding status*Yr1 + Yrs2-4)	3	Survival varies by breeders and non-breeders in Year 1 and is constant thereafter
Transmitter effect models		
S(Yr1 + Yrs2-4)	2	Survival in Year 1 varies from years thereafter
S(Yr1*Trend + Yrs2-4)	3	Survival varies in a linear monthly trend during Year 1 and is constant thereafter
S(Mo1-2 + Mo3-12 + Yrs2-4)	3	Survival varies by Months 1-2 and Months 3-12 and is constant thereafter
S(Yr1*Trend + Yr)	5	Survival varies in a linear monthly trend during Year 1 and by year thereafter
S(Mo1-2 + Mo3-12 + Yr)	5	Survival varies by Months 1-2 and Months 3-12 and by year thereafter
Season * Year models		
S(Migration*Yr1 + Yrs2-4)	3	Survival during migration seasons (Autumn,Spring) varies from sedentary seasons (Winter, Breed) in Year 1 and is constant thereafter
S(Autumn*Yr1 + Yrs2-4)	3	Survival during Autumn varies from other seasons (Spring, Winter, Breed) in Year 1 and is constant thereafter
S(Season*Yr1 + Yrs2-4)	5	Survival varies by 4 seasons (Spring + Autumn + Winter + Breed) in Year 1 and is constant thereafter
S(Migration*Yr1 + Yr)	5	Survival during migration seasons (Autumn, Spring) varies from sedentary seasons (Winter, Breed) in Year 1 and by year thereafter
S(Autumn*Yr1 + Yr)	5	Survival during Autumn varies from other seasons (Spring,Winter,Breed) in Year 1 and by year thereafter
S(Season*Yr1 + Yr)	7	Survival varies by 4 seasons (Spring + Autumn + Winter + Breed) in Year 1 and by year thereafter

We considered a model in which survival varied linearly relative to mean annual migration distance for each breeding area. To account for additional sources of variation in survival such as those resulting from differences in migration routes, wintering locations, or factors specific to the breeding grounds (Table 1, [41]), we examined a breeding area-specific model, and 2 simplified location models. Tundra swans in North America are managed as 2 distinct populations, the Western Population (WP) which winters on the Pacific coast of the United States and Canada and the Eastern Population (EP) which winters along the eastern seaboard of the United States. In our first simplified location model, we assessed potential differences in survival between management populations by constraining survival among birds from the WP (LAP, BBL, YKD and KSL) separate from the EP (CRD). Of the WP birds in our marked sample, LAP birds are unique in that many are mostly nonmigratory and winter near their breeding grounds on the Alaska Peninsula [45] (Table 1). YKD and KSL birds first migrate eastward and then south through western Canada, Montana, and Utah into California ([27, 41]; Fig. 1). BBL birds migrate more westerly than YKD and KSL birds, and winter predominantly in the Pacific Northwest [41]. CRD birds had the longest migration, as they traversed through north central Canada, then flew across the mid-continent northern prairies before continuing eastward to winter along the east coast [28, 41]; (Fig. 1). In our final location model, we assessed variation in survival relative to wintering area by considering a model that grouped birds from the three migratory WP breeding areas (BBL, YKD, KSL) and estimated survival unique to the non-migratory WP location (LAP), and the breeding area from the EP population (CRD) (Table 2).

To assess seasonal variation in MSP, months were grouped by periods that approximated varying life-history components and differential risks to survival. June through August were breeding months when successful breeders reared young and failed or non-breeders congregated in molting flocks; September through December represented autumn staging and migration when birds were exposed to hunting pressure and the physiological stress of migration; January and February were winter months during which birds remained at localized areas; and March through May represented spring migration and return to the breeding grounds. A season-specific model was considered in which survival was estimated separately for each of the 4 seasons. Because survival may be lowest during fall when swans are exposed to hunting pressure and risks associated with migration, a model was considered where survival in fall months was estimated separately from the remaining months. A third season model assessed variation in survival of migration months (spring and autumn) versus non-migration months (winter and breeding) under the hypothesis that mortality risks were higher during migration than while birds were generally sedentary (Table 2).

A year-specific model was considered to account for annual variation in survival and a number of models in which the first year post-release (Aug, 2008–July, 2009) was estimated separately from remaining years (Aug, 2009–July, 2012; Table 2). These included 3 models to assess potential deleterious effects associated with surgery and implantation of the transmitters, and 6 models in which various season models were combined with models of annual variation. Mortality risks associated with capture and transmitter implantation were expected to be highest immediately following surgery, and decrease thereafter. To assess this hypothesis, a model was considered where survival was estimated separately for months 1–2 post release and months 3–12 post release, a model where survival increased linearly during the first 12 months post release, and a model where survival in the first year was different than survival in years 2–4.

Subadult swans typically have lower survival than adults [46, 47], therefore age effects were assessed by considering a model where survival was age-specific in the first year post-release (Table 2). Likewise, the effect of breeding status in the first year post-release was assessed by estimating survival separately for birds captured with cygnets or on breeding territories (breeders) and those from molting flocks (non-breeders). Breeders were expected to have higher survival than non-breeders because breeders are generally older birds and have thus already survived multiple annual cycles.

Relative support among models was assessed using Akaike’s Information Criterion corrected for sample size (AIC_c_) and model weights (*w*_*i*_; [48]). To avoid selecting a model with uninformative parameters, we considered models with one additional parameter competitive only if their AIC_c_ values were lower than the simpler model [48, 49]. Estimates of MSP were back-transformed from the logit link and the delta method was used to calculate associated variances. Estimates of annual survival were calculated as the product of 12 MSPs, corresponding to August through July.

### Estimates of productivity

Estimates of productivity (% young in winter flocks) of tundra swans from the different breeding areas was obtained from the Management Plan for Western Population Tundra Swans [50], the Management Plan for Eastern Population Tundra Swans [51], and from field studies conducted on the Lower Alaska Peninsula (C. Dau and K. Sowl, unpubl. data). Productivity estimates for the WP collected in Utah were presumed to represent birds from western Alaska (YKD and KSL), whereas productivity estimates from Washington were presumed to represent birds from the Bristol Bay Lowlands, based on distribution of satellite-marked and neck-banded tundra swans ([41]; C. Ely et al. unpubl. data).

## Results

### Characteristics of marked birds

Ten birds were implanted with PTTs at each of the five breeding areas (Fig. 1). Twenty-five implanted birds were categorized as breeders or in territorial pairs, and 25 were determined to be non-breeders. Of the 25 birds that were breeders or on territories, 21 were ASY females and four were ASY males (from the YKD), whereas of the 25 non-breeders, 17 were ASY females, six were SY females, and two were SY males. None of the implanted birds tested positive for LPAI viruses [52].

### Migration distance

**Fig. 2:**
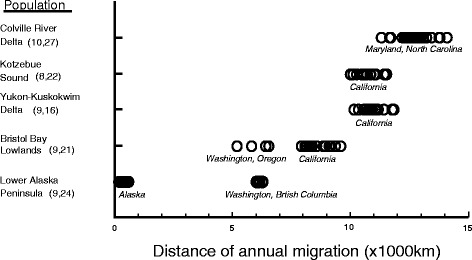
Distances moved during migration by satellite-transmittered tundra swans from five different breeding areas in Alaska. Values in parentheses are the number of different birds completing at least one full (autumn + spring) migration, followed by the total number of complete migrations documented for each population. Site names inside the plot indicate winter location

There was significant variation across breeding populations with respect to migration routes and distances travelled between breeding and wintering areas with CRD>YKD,KSL>BBL>LAP (F_3,41_ = 516.55, *P* <0.0001; and *P* <0.0001 for all pairwise comparisons – Table 1, Fig. 2). Intra-population variance in migration distance was particularly high for LAP swans, as most birds did not migrate away from Alaska, and for BBL swans which used two different terminal wintering areas that were at different distances from the breeding area (Figs. 1 and 2). The time swans spent on the different breeding areas also varied significantly among populations, with LAP>BBL>YKD,KSL>CRD (F_3,40_ = 327.16, *P* <0.0001; and *P* <0.0001 for all pairwise comparisons – Table 1). The mean time spent at each breeding area (*n* = 5) was strongly negatively correlated with mean distance travelled on migration (*r* = −0.986).

### Survival

**Fig. 3:**
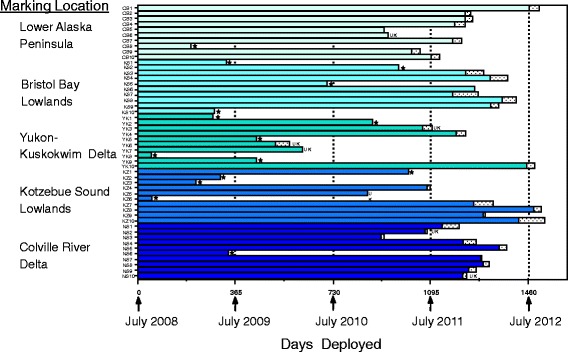
Histories of 50 tundra swans implanted with satellite transmitters (PTTs) at 5 different breeding areas in Alaska in 2008. Stippled horizontal bars indicate number of days swans were known alive based solely on body temperature information. * designates birds assumed to be dead based on low body temperatures just before or at the time of final transmission. UK indicates a bird whose fate was in doubt, as the PTT battery voltage was not low when transmission ceased

The encounter histories of 50 PTT-marked tundra swans is presented in Fig. 3. Of the eight birds in our sample that were SY at the time of marking, none died in the first year post-release. Of 15 definitive mortalities, 9 occurred in the first year post-marking, three occurred in each of the 2^nd^ and 3^rd^ years, and none occurred in year 4. The majority of transmitter failures occurred in year 4 (Table 3); four transmitters were still operating at the conclusion of the study. There were seven transmitters for which fate was not discernible (i.e., discrepancies) because the body temperature and battery voltage were both within normal levels upon the final transmission event. These occurred in year 2 (*n* = 2), year 3 (*n* = 4), and year 4 (*n* = 1) (Table 3). No birds died in the first month following release. Fifty percent (25 of 50) of the implanted swans were either with cygnets or on territories, and of the 15 definite mortalities during the course of the study, 10 were swans designated as breeders at the time of capture. Five of the ten mortalities of breeders occurred in the first year after marking.

**Table 3:** Fate of tundra swans marked with abdominally implanted satellite transmitters in Alaska in 2008

		Fate of marked birds			
Year	Interval	Survived	Died	Transmitter failures	Unknown
1	(Aug-2008–Jul-2009)	41	9	0	0
2	(Aug-2009–Jul-2010)	36	3	0	2
3	(Aug-2010–Jul-2011)	25	3	4	4
4	(Aug-2011–Jul-2012)	4	0	20	1

Swans with unknown fates (i.e. “discrepancies”) had transmitters that reported normal body temperature and normal battery voltage in their last transmission. All mortalities the first year were ASY birds

In the first analysis, encounter histories of discrepancy birds were censored and therefore estimated survival was based on 15 mortalities. Two models in the initial suite of 22 models received substantial support; these included the ‘wintering area’ model in which survival varied by LAP, CRD and the grouping of BBL, YKD, and KSL, and an ‘age’ model that constrained Yr1 separate from Yrs2-4 and contained an age-effect in Yr1 (Table 4). Combining variables from the two supported models resulted in 6 additional models that assessed variation in survival by wintering location, year, and age (Table 5). The top approximating model from the final model set (*w* = 0.51) predicted differential survival in Yr1 separate from Yrs2-4, an age effect in Yr1, and variation in survival by wintering area for Yrs2-4 (Table 5). We did not find support for variation in survival relative to migration distance (ΔAIC_c_ = 4.14; *w* = 0.02; ${\widehat{\beta}}_{\mathrm{mig}\;\mathrm{dist}}=-0.0001;85\%\;\mathrm{C}\mathrm{I}:-0.00020,0.00007$), season, or breeding status (Table 4). MSP in Yr1 for ASY swans from all locations was 0.981 (SE = 0.006). Monthly survival in Yrs2–4 was 0.989 (SE = 0.005) for BBL, YKD, and KSL birds. No SY swans died in the first year, and no birds from LAP or CRD died in Yrs2-4, so MSP was 1.0 for each of these groups.

**Table 4:** Model selection results for estimating monthly survival probability (MSP) of PTT-implanted tundra swans from Alaska, USA, 2008–2012. Results include 22 models from the a priori model set and are based on a version of the data containing 15 mortalities (see text). Models are ranked based on Akaike’s Information Criterion adjusted for small sample sizes (AICc) and model weight (*w*_i_). K = number of model parameters; AICc of top model = 168.19

MSP model	K	ΔAICc	*w*_i_	Deviance
Wintering area	3	0.00	0.19	162.17
Age * Yr1 + Yrs2-4	3	0.09	0.18	162.26
Yr1 + Yrs2-4	2	1.48	0.09	165.66
Yr1 * Trend + Yrs2-4	3	1.85	0.07	164.02
Management population	2	2.08	0.07	166.26
Autumn * Yr1 + Yrs2-4	3	2.83	0.05	165.01
Constant	1	3.10	0.04	169.28
Breeding area	5	3.19	0.04	161.34
Mo1-2 + Mo3-12 + Yrs2-4	3	3.38	0.03	165.55
Breeding Status * Yr1 + Yrs2-4	3	3.39	0.03	165.56
Yr	4	3.45	0.03	163.61
Migration * Yr1 + Yrs2-4	3	3.45	0.03	165.62
Yr1 * Trend + Yr	5	3.82	0.03	161.97
Migration distance	2	4.14	0.02	168.32
Migration	2	4.80	0.02	168.98
Autumn * Yr1 + Yr	5	4.80	0.02	162.95
Autumn	2	5.02	0.02	169.20
Mo1-2 + Mo3-12 + Yr	5	5.34	0.01	163.49
Migration * Yr1 + Yr	5	5.42	0.01	163.57
Season * Yr1 + Yrs2-4	5	6.64	0.01	164.79
Season	4	8.04	0.00	168.21
Season * Yr1 + Yr	7	8.62	0.00	162.74

**Table 5:** Second-stage model selection results for estimating monthly survival probability (MSP) of PTT-implanted tundra swans from Alaska, USA, 2008–2012. Results are based on a version of the data containing 15 mortalities (discrepancies censored), and include the top two approximating models from the a priori model set (in bold font), and six additional models containing combinations of parameters from these two models. Models are ranked based on Akaike’s Information Criterion adjusted for small sample sizes (AICc) and model weight (*w*_i_) that are re-standardized considering only these 8 models. K = number of model parameters; AICc of top model = 164.61

MSP model	K	ΔAICc	*w*_i_	Deviance
(Age * Yr1) + (Yrs2-4 * Wintering area)	5	0.00	0.51	154.58
(Age * Yr1 + Wintering area) + (Yrs2-4 * Wintering area)	7	2.60	0.14	153.14
Age * Yr1 + Yrs2-4 + Wintering area	6	3.39	0.09	155.95
**Wintering area**	**3**	**3.57**	**0.09**	**162.17**
Age * Yr1 + Yrs2-4	**3**	**3.66**	**0.08**	**162.26**
(Age * Yr1 + Yrs2-4) * Wintering area	8	4.62	0.05	153.14
(Age * Yr1) + (Yrs2-4 + Wintering area)	5	6.25	0.02	160.83
Age * Yr1 * Wintering area + Yrs2-4	6	8.27	0.01	160.83

**Fig. 4:**
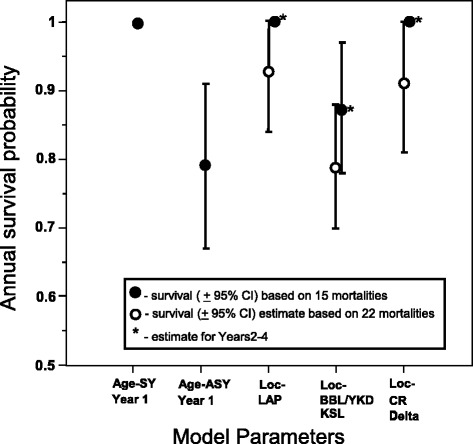
Annual survival probabilities of satellite-implanted tundra swans based on 15 mortalities (*solid circles*) or 22 mortalities (*open circles*). Age and year were supported parameters for the 15-mortality model while only location was supported in the 22-mortality model

For the second analysis, a more liberal approach was employed to discern mortalities, and 22 birds were considered to have died. The best supported model (*w* = 0.17) constrained survival by wintering area (Table 6). The ‘migration distance’ model received limited support (ΔAIC_c_ = 1.15; *w* = 0.10) but was not supported over the ‘constant’ model (ΔAIC_c_ = 1.01 *w* = 0.11), and the migration distance effect was equivocal (${\widehat{\beta}}_{\mathrm{mig}\;\mathrm{dist}}=-0.00008;85\%\;\mathrm{C}\mathrm{I}:-0.00016,0.00001$). There was little support for variation in survival relative to transmitter effects, season, breeding status, or year, so additional models were not considered. Monthly survival of swans from LAP was 0.994 (SE = 0.004), for CRD was 0.992 (SE = 0.005) and for BBL, YKD, and KSL was 0.980 (SE = 0.005). True survival of our PTT-marked birds is likely bracketed between estimates from conservative and liberal approaches (Fig. 4).

**Table 6:** Model selection results for estimating monthly survival probability (MSP) of PTT-implanted tundra swans from Alaska, USA, 2008–2012. Results include 22 models from the a priori model set and are based on a version of the data containing 22 mortalities (discrepancies considered mortalities). Models are ranked based on Akaike’s Information Criterion adjusted for small sample sizes (AICc) and model weight (*w*_i_). K = number of model parameters; AICc of top model = 232.52

MSP model	K	ΔAICc	*w*_i_	Deviance
Wintering area	3	0.00	0.18	226.50
Breeding area	5	0.71	0.13	223.19
Constant	1	1.01	0.11	231.53
Migration distance	2	1.15	0.10	229.66
Age * Yr1 + Yrs2-4	3	1.37	0.09	227.87
Management population	2	1.70	0.08	230.21
Autumn	2	2.76	0.05	231.27
Yr1 + Yrs2-4	2	2.77	0.05	231.28
Migration	2	3.02	0.04	231.53
Yr1 * Trend + Yrs2-4	3	3.13	0.04	229.63
Autumn * Yr1 + Yrs2-4	3	4.12	0.02	230.62
Mo1-2 + Mo3-12 + Yrs2-4	3	4.66	0.02	231.16
Breeding status * Yr1 + Yrs2-4	3	4.67	0.02	231.17
Migration * Yr1 + Yrs2-4	3	4.73	0.02	231.23
Yr	4	5.29	0.01	229.78
Yr1 * Trend + Yr	5	5.66	0.01	228.14
Autumn * Yr1 + Yr	5	6.64	0.01	229.12
Season	4	6.79	0.01	231.28
Mo1-2 + Mo3-12 + Yr	5	7.19	0.01	229.67
Migration * Yr1 + Yr	5	7.26	0.00	229.74
Season * Yr1 + Yrs2-4	5	7.93	0.00	230.41
Season * Yr1 + Yr	7	10.47	0.00	228.91

### Timing of mortality

There was no support found for models that included seasonal variation in survival, as mortalities of implanted swans were nearly equally distributed across different phases of the annual cycle (Fig. 3; Table 4). Because of the different migration chronologies across breeding areas, a combination of date and location was used to categorize “season” of mortality. Of the 15 PTT-implanted swans that we are confident died during the study (based on low body temperature), 7 (46.7 %) died during summer (on breeding areas), 5 (33.3 %) died during migration (while in transit between breeding and wintering areas; 3 in spring and 2 in autumn), and 3 (20 %) died during winter (while at terminal southern sites). Of the summer mortalities, one occurred before nest initiation (26 May 2009 in Kotzebue Sound Lowlands), two during the incubation period (11 June 2009 and 13 June 2010 in the Bristol Bay Lowlands), two during the early brood rearing period (12 July on the Y-K Delta and 13 July on the Colville River Delta), and two just before autumn migration (16 September 2008 on the Y-K Delta, and 16 September 2008 in the Kotzebue Sound Lowlands; Fig. 3).

### Population-specific productivity

**Fig. 5:**
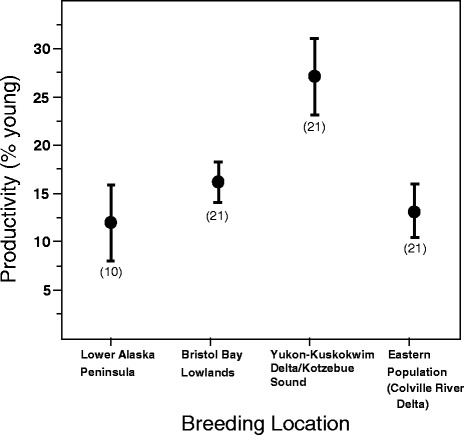
Productivity of tundra swans from 4 breeding area in Alaska, as represented by percentage of immature (hatch year) birds observed in winter flocks. Data for the Lower Alaska Peninsula are from 1978 to 2006, non-inclusive, whereas estimates from the other sites are for 1980–2000 (see Methods). Eastern population swans are represented by Colville River Delta swans. Values in parentheses are the number of years with productivity estimates

Productivity, determined by the proportion of young swans observed in winter flocks, varied significantly among swans from the different breeding areas (Fig. 5; F_3,72_ = 20.62, *P* <0.0001). Tundra swans from western Alaska (YKD & KSL) had higher productivity (27.0 %) than swans from the other areas (12.0 % for LAP, 16.2 % for BBL, and 13.3 % for CRD). Productivity estimates for BBL swans may be slightly underestimated as age ratio counts in Washington were conducted throughout the winter, rather than in autumn only as with other populations, and therefore include over-winter mortality of juveniles.

## Discussion

### Influence of migration distance on mortality

Migration distance was not included in any of the top-supported survival models (Table 4), despite the tremendous variation among populations in distances travelled (Fig. 2). While this result might seem somewhat surprising given the great range of migration distances (and concomitant energy investment) among breeding areas, it is more understandable if one considers the energetic burden of migration in terms of daily energy expenditure. Tundra swans are known to be slow migrants, and can take up to several months to move between wintering and breeding areas [27–29, 53]. If the energetic cost of migration is spread out across a long time period, then the daily or weekly burden of energy intake is greatly diminished and migrants can spend more time in non-foraging activities such as predator detection and pair and family maintenance. Prolonging migration over several months may also benefit tundra swans indirectly if such a tactic releases them from energetic or behavioural constraints such as hyperphagia and rapid weight gain, which several authors have speculated increases susceptibility to predation relative to leaner birds [3, 54, 55]. A loss of agility, even in tundra swans (whose large body size provides protection from many predators), could prove decisive, as they rely on agility to avoid eagles and other large predators, which are common on staging areas across North America. However, Nearctic tundra swans are considered to be income breeders (Nolet 2006), and therefor likely do not undergo the great increases in spring body mass as other large-bodied arctic-nesting waterfowl that are capital breeders. As such they may be relinquished from the burden of carrying large energy stores and the concomitant increased risk of predation.

Few other studies have simultaneously estimated magnitude and/or timing of mortality relative to flight distance in birds, and of the studies conducted, there has not been a consensus on the relationship between migration distance and mortality. Sandercock and Jaramillo [22] used a modern analytical approach to determine survival rates of several passerine species relative to migration distance and found no relationship, nor did Souchay et al. [56] who studied two subpopulations of greater snow geese (*A. caerulescens atlanticus*) that migrated different distances. In contrast, Gillis et al. [57] found that resident dippers (*Cinclus mexicanus*) had lower annual survival rates than migrants, and Varner and Eicholz [58] found a positive relationship between survival and migration distance for subadult (but not adult) trumpeter swans (*Cygnus buccinator*). A similar age-dependent relationship between migration distance and survival has been found for greater flamingos (*Phoenicopterus roseus*; [59]), with young birds showing a negative relationship and older birds a positive relationship, the latter of which was attributed to the better body condition of adult birds migrating to a more suitable, but distant, wintering area. Alves et al. [60] reported that populations of Icelandic black-tailed godwits (*Limosa limosa islandica*) that wintered in milder climates fared better than populations wintering in a harsher environment, regardless of migration distance, while Lok et al. [61] found that Eurasian spoonbills (*Platalea leucorodia* – a large-bodied waterbird) that migrated the furthest were the least likely to survive, which they attributed, in part, to constraints of tradition, or “migratory tendency”. The latter finding highlights the difficulty in finding generality in migration strategies across species with different life history traits, especially if behavioral attributes such as migration tradition, are unaccounted for.

### Transmitter effects

Attaching a transmitter to an animal can cause mortality directly due to physical encumbrance or indirectly by negatively affecting behaviour that could increase vulnerability to predators or limit foraging. Many negative impacts of external transmitters can be overcome with transmitter implantation, but internal devices and the surgical procedures necessary to implant them might also reduce survival. The 2–4 weeks following surgical implantation are considered by many to be the time when surgery-related mortality would most likely occur [32, 62]. None of the tundra swans in this study died during this post-operative period, suggesting that acute effects of implantations were minimal. However, there may have been delayed (>2 months post release) effects of handling and implantation as the version of encounter histories that used a conservative approach (i.e. 15 mortalities) contained a disproportionate number of mortalities in the first year post-release (9 of 15). Also, the estimate of annual survival for the year immediately following release (0.79) was lower than estimates for the subsequent 3 years (range: 0.87–1.0). However, several factors indicate that transmitter effects were likely minimal, and if present, were likely limited to the first year post release, as 1) survival in the first 2 months was not lower than the following 10 months; 2) MSP during the first year post-release did not increase linearly through time; and 3) mortalities were distributed evenly throughout the first year post-release.

Previous studies of large waterfowl, including common eiders (*Somateria mollisma),* have demonstrated that implantation of transmitters can lead to first year mortality, affect behaviour, and influence dive performance [63, 64]. However, the birds in these studies were likely adversely affected by transmitter implantation during the nesting period [63], and by the effects of organ compression during diving [64]. In contrast, in a species morphologically and behaviourally more similar to swans, Hupp et al. [33] studied the effects of implanted radios on the behaviour and survival of Canada geese (*Branta canadensis*) and found very few deleterious effects, with just 1 % of overall mortality attributed to transmitter implantation.

### Timing of mortality

The finding that mortalities of swans in this study were evenly distributed throughout the annual cycle is in contrast to the findings of other studies that have reported declines in survival during specific times of the year. Although Gauthier et al. [51] reported adult survival rates in greater snow geese were highest during periods of migration and reproduction, most studies of large-bodied migratory birds have shown survival is lower during periods of migration, including barnacle geese (*Branta leucopsis*; [65]), greater white-fronted geese (*Anser albifrons*; [66]), and emperor geese (*Anser canagicus*; [44]). In perhaps the most extensive study of the timing of mortality events, Klaassen et al. [13] reported that mortalities of 69 satellite-tracked raptors occurred throughout the year, but that mortality was highest during periods of migration. Hence, despite our finding here, there is ample evidence that the migration period is costly to many species of long-lived migratory birds. However, even in species for which costs can not be directly linked to the period of migration, it is possible that the cumulative costs of migration may be realized outside periods of travel, whereby deficits incurred during migration may not be manifest until a later date, and hence “carried over” [67]. Such carry over effects could be in the form of nutrient deficits, delayed responses to pathogen exposure, or to decreased survival due to a breakdown in pair bonds and family structure, especially in species such as swans and geese with complex multi-generational social structures. Social structure is likely most labile in geese and swans while they are in large concentrations on staging areas when disturbance by predators and humans is likely magnified [68].

### Survival estimates compared to other studies

Our estimates of annual survival for swans marked on the Lower Alaska Peninsula ranged from 0.93 to 1.0. This is considerably higher than the mean apparent annual survival estimate of 0.61 during the years 1978–1989 for neck-collared swans at the same location [47]. Meixell et al. [47] suggested the low and variable estimates of apparent annual survival for LAP swans may have resulted from high and variable rates of permanent emigration. Of the 10 swans we marked on the LAP, only 2 migrated out of Alaska during the winter, and both individuals returned the same year. A high proportion of neck banded swans from the LAP were detected on wintering areas each year (C. Ely et al. unpubl. data), which demonstrates heterogeneity in the migration tendencies of LAP swans, although it does not necessarily establish them as an open population.

High annual survival in swans as found in this study has also been noted by other investigators. In a cross-species comparison, Bart, Earnst and Bacon [69] found that migratory swan species, including tundra, Bewick’s (*C. c. bewickii*), whooper (*C. cygnus*), and trumpeter (*C. buccinator*), had higher annual adult survival rates (near 90 %), than non-migratory adult swans, including mute (*C. olor*), black (*C. atratus*), and black-necked (*C. melanocoryphu*s) which generally had annual survival rates of 80–85 %.

### Possible sources of variation in survival

For both versions of the data used in estimation of survival the top models differentiated survival by wintering location, predicting that swans with winter ranges along the Pacific coast had lower survival than those that wintered on the eastern seaboard and the relatively sedentary swans from the lower Alaska Peninsula. There are many factors related to the distribution of avian populations that may affect survival [70]. Hunter harvest is known to limit populations of large-bodied Anatidae [71] and could potentially impact tundra swan populations, although none of our PTT-marked birds were reported to have been harvested. This is not too surprising as federal and state/provincial agencies generally restrict harvest of tundra swans compared to other waterfowl, and on average <5 % of wintering populations is harvested in any given year [72, 73]. Also, it is generally presumed that survival and susceptibility to predation in particular is influenced by overall health, including parasite load, disease prevalence, exposure to contaminants, and immunocompetence [74]. We cannot completely rule out the possible impacts of these variables on survival, however, in a co-investigation of blood parasites using some of the same birds as this study, Ramey et al. [34] did not find a relationship between migration distance and prevalence of blood parasites, as swans from the population with the highest infection rate (BBL) migrated only a moderate distance compared to birds with lower infection rates that flew shorter (LAP) and longer (CRD) distances. Also, none of the swans in this study were infected with LPAI viruses at the time of capture, or had blood lead levels high enough to cause adverse effects [41]. It is also possible that the higher mortality of BBL, YKD, and KSL swans is related to their winter sympatry (Fig. 1), but the cause(s) of their lower survival is unclear.

### Time allocation and life history trade-offs

Our top survival model did not include migration distance as a parameter, and since migration distance was equitable with time spent on breeding areas (*r*^2^ = 0.97; Table 1); the latter, and its reciprocal (days spent on non-breeding areas) were also not predictive of survival probability. As such, tundra swan populations overall did not adhere to the time allocation hypothesis, at least in terms of factors influencing survival. There are several aspects of the life history of tundra swans which may help explain why this might be the case. Earlier in the discussion we considered why species that migrate very slowly, such as tundra swans, may not be impacted by the energetic cost of migration. The large body size of tundra swans is another life history attribute that sets them apart from many other species; because of thermoregulation considerations related to body mass, tundra swans may not benefit as much from travelling further to mild wintering grounds as the smaller passerines studied by Greenberg [15]. In fact, the tundra swans population that migrated the furthest (CRD) did not winter in a milder climate than the other populations, as mid-winter temperatures (January) in coastal North Carolina (Nags Head; 2.2 °C) are colder than in the Central Valley of California (Stockton; 3.3 °C) where KSL and YKD swans winter.

Although Alaska-breeding tundra swans did not strictly adhere to the predictions of the time allocation hypothesis, there were patterns in demographic variables across the populations that are worth noting, the most obvious of which was the inverse relationship between survival and productivity, with CRD and LAP swans having relatively high survival and low productivity as compared to YKD/KSL swans which had relatively low survival, but high productivity (Figs. 4 and 5). This is worthy of note, as in stable populations (which these populations are [72, 73]), survival and reproduction are off-setting [75], but empirical data supporting this tenet at the population level is quite rare. The low productivity of BBL swans, despite a relatively low survival rate, may be related to higher nest predation rates at lower latitudes [17], saturated breeding habitats leading to an overabundance of non-breeders, or to other factors intrinsic to the Alaska Peninsula such as a high brown bear [*Ursus arctos*] population.

Swans are long-lived birds, and the fact that migration distance was not a strong predictor of survival or that demographic parameters of the different populations do not conform to predictions of the time allocation hypothesis, may be related to annual variation in selective forces and hence underlying deficiencies in the temporal scale of our research or the number of birds we monitored. Future research should focus on breeding-area-specific factors influencing annual variation in survival and productivity, and on monitoring the long-term reproductive success of individual birds relative to social status. Insights into factors influencing survival are to be gained from research leading to a better understanding of the trade-offs between income and capital breeding strategies among these populations of very slow migrants.

## Conclusions

Migration distance of satellite-transmittered tundra swans from five different breeding areas in Alaska varied among populations from 1020 to 12720 km annually, but distance travelled was not a strong predictor of variation in survival. Mortalities were not more common during periods of migration, but occurred throughout the year, further indicating that long distance migration may not negatively affect survival of tundra swans. Productivity varied among breeding populations and was inversely correlated with survival in four of the five populations. The absence of a uniform relationship between the amount of time allocated for breeding and nonbreeding activities relative to productivity and survival indicates that temporal investment does not greatly influence demographic patterns in tundra swans. Our findings suggest that tundra swan populations are not encumbered demographically by diverse migration strategies; a flexibility and predisposition that may prepare them for climate-induced changes in migration patterns and habitat use. Additional studies of metapopulations of wild birds are necessary to better understand the interrelationships between migration strategies and time investment and their impact on survival and productivity.

### Availability of supporting data

The location and sensor data for the PTT-implanted tundra swans supporting the results of this article are available in Movebank (https://www.movebank.org) and are available upon request. Maps showing movement of individual birds can also be accessed via the Alaska Science Centre website: http://alaska.usgs.gov/science/biology/avian_influenza/TUSW/index.php#map.
